# Transcriptomic and targeted metabolomic insights into carotenoid-mediated color formation in sorghum grains

**DOI:** 10.3389/fpls.2025.1724008

**Published:** 2026-01-20

**Authors:** Wenzhen Li, Yanqing Ding, Ning Cao, Bin Cheng, Jianxia Xu, Xu Gao, Ruoruo Wang, Kuiyin Li, Liyi Zhang

**Affiliations:** 1 Guizhou Key Laboratory of Biology and Breeding for Specialty Crops, Guizhou Institute of Upland Crops, Guizhou Academy of Agricultural Sciences, Guiyang, China; 2 Plant Conservation Technology Center, Guizhou Key Laboratory of Agricultural Biotechnology, Biotechnology Institute of Guizhou Province, Guizhou Academy of Agricultural Sciences, Guiyang, China; 3 College of Agriculture, Anshun University, Anshun, China

**Keywords:** sorghum, grain color, carotenoid accumulation, metabolomics, transcriptomics

## Abstract

**Introduction:**

Sorghum is a major staple crop in semi-arid regions; however, its generally low grain carotenoid content limits its potential contribution to alleviating vitamin A deficiency. Elucidating the regulatory mechanisms underlying carotenoid accumulation is therefore essential for the nutritional improvement of sorghum.

**Methods:**

Five sorghum varieties with distinct grain colors (white, gray, yellow, red, and black) were analyzed using integrated targeted carotenoid metabolomic and transcriptomic approaches to characterize carotenoid composition and its molecular regulation.

**Results and Discussion:**

A total of 37 carotenoid compounds were identified across the five sorghum varieties, with lutein as the predominant component. The yellow-grained variety exhibited the highest total carotenoid content (16.79 ± 0.61 mg/g), whereas the red-grained variety showed the lowest overall content but accumulated several unique carotenoids. Transcriptomic analysis identified nine key differentially expressed genes involved in carotenoid metabolism, including genes associated with precursor supply (geranylgeranyl pyrophosphate synthase, GGPPS), core carotenoid biosynthesis (15-cis-phytoene desaturase, PDS), xanthophyll modification (cytochrome P450 716A1, CYP716A1), and carotenoid catabolism (9-cis-epoxycarotenoid cleavage dioxygenase 5, NCED5). These genes displayed distinct expression patterns among varieties, indicating coordinated regulation of carotenoid biosynthesis and degradation. Correlation analysis further revealed that PDS and CYP716A1 were significantly associated with the accumulation of β-carotene, lutein, and zeaxanthin. Collectively, these findings demonstrate a transcriptionally regulated carotenoid metabolic network in sorghum and indicate that grain color alone does not reliably predict carotenoid composition, as other pigments such as anthocyanins and tannins also contribute to grain coloration. PDS and CYP716A1 are therefore identified as promising targets for carotenoid biofortification and for the development of nutritionally enhanced sorghum varieties.

## 1 Introduction

Vitamin A deficiency (VAD) is one of the most prevalent and serious micronutrient deficiencies worldwide, with a particularly widespread impact in developing countries. According to statistics from the World Health Organization, over 250 million preschool children and millions of women of childbearing age worldwide are affected by VAD (Yamaguchi, 2012; Ndolo and Beta, 2013). VAD not only leads to night blindness and impaired immune function but can be life-threatening in severe cases (Surman et al., 2020). This problem is especially prominent in regions where grains are the staple food, as grains generally have low levels of natural vitamin A precursors (carotenoids), which are insufficient to meet human nutritional needs (Cruet-Burgos and Rhodes, 2023). Sorghum (*Sorghum bicolor (L.) Moench*), as a core coarse grain crop in semi-arid areas, plays a crucial role in ensuring food security in sub-Saharan Africa and South Asia (Félix et al., 2018; Hadebe et al., 2021). However, common sorghum varieties have relatively low carotenoid content (Kean et al., 2007). Enhancing the accumulation of carotenoids with vitamin A activity (such as β-carotene and β-cryptoxanthin) in sorghum grains through genetic improvement not only provides a sustainable source of vitamin A for local populations but also improves the nutritional quality of sorghum. This holds significant practical importance for enhancing public health in these regions (Aluru et al., 2008; Ofori et al., 2022).

Sorghum grains exhibit a rich diversity of colors, including white, yellow, red, brown, and black phenotypes. These color differences are mainly determined by the types and contents of pigment compounds (Shrestha et al., 2025). Carotenoids are the core pigments in yellow and orange grains (Salas Fernandez et al., 2009; McDowell et al., 2024), while phenolic substances such as anthocyanins and tannins dominate the coloring of red and brown grains (Li et al., 2021; Santos et al., 2024). Although previous studies have confirmed an association between sorghum grain color and carotenoid content, the mechanisms underlying carotenoid biosynthesis and accumulation have not been systematically elucidated. The specific composition and content differences of carotenoids in sorghum grains of different colors (e.g., white, yellow, red) have not been systematically quantified. In particular, the distribution pattern of carotenoids with vitamin A activity in grains of different colors remains unclear, and the internal molecular regulatory mechanisms responsible for these differences are still in the stage of fragmented research (Dykes et al., 2011). These factors greatly limit the efficiency of precisely improving sorghum carotenoid content through molecular breeding (Blanco et al., 2011).

Carotenoid biosynthesis is a complex multi-step metabolic process. In plants, it is catalyzed by a series of enzymes encoded by nuclear genes in plastids, and this process is coordinately regulated by multiple genes (Ampomah-Dwamena et al., 2009; Quinlan et al., 2012; Chen et al., 2024). Currently, only a few key genes, such as PSY and ZEP, have been preliminarily associated with carotenoid synthesis in sorghum grains (Cruet-Burgos and Rhodes, 2023; McDowell et al., 2024). The genes involved in carotenoid biosynthesis in sorghum grains show dynamic expression patterns during grain development. Most carotenoid synthesis genes are highly expressed in the early stage of grain development (14 days after pollination), while the expression of carotenoid degradation genes and some branch-point genes are higher in the late development stage (Cruet-Burgos and Rhodes, 2023). Studies have found that the regulation of this pathway involves multiple levels, including transcriptional level (expression of key enzyme genes), post-transcriptional level (modification of enzyme activity), and metabolic flux distribution (competition among branch enzymes). However, a single omics technology is insufficient to fully reveal its regulatory network (Zheng et al., 2023; Yu et al., 2025). The integrated analysis strategy combining carotenoid – targeted metabolomics and transcriptomics has become a mainstream approach for deciphering the mechanisms of plant secondary metabolite biosynthesis, providing a systematic perspective for clarifying complex metabolic networks (Jia et al., 2022; Zhang et al., 2023).

This study aims to systematically clarify the metabolic and transcriptional regulatory basis underlying the differences in carotenoid accumulation in sorghum grains of different colors. High-performance liquid chromatography (HPLC) and liquid chromatography-mass spectrometry (LC-MS) techniques were employed to quantitatively analyze the composition and distribution of carotenoids in grains of different colors. Combined with RNA-seq, differentially expressed genes in the carotenoid biosynthesis pathway were screened. Furthermore, an integrated analysis of carotenoid – targeted metabolome and transcriptome was conducted to construct a core regulatory module, and the functions of key genes were analyzed in depth. This study not only fills the research gap in the regulatory mechanism of carotenoids in sorghum grains of different colors but also provides key gene markers and theoretical basis for carotenoid biofortification breeding of sorghum. It is of great significance for synergistically improving the nutritional quality and appearance quality of crops and alleviating the problem of vitamin A deficiency.

## 2 Materials and methods

### 2.1 Experimental materials

The grain sorghum varieties utilized in this study, namely BTx623 with white grain color(W), P898012 with gray grain color(G), Tx430 with yellow grain color(Y), HYZ with red grain color(R), and BYQ with black grain color(B), were collected and maintained by the Institute of Dryland Crops at the Guizhou Academy of Agricultural Sciences. These varieties were cultivated at the institute’s experimental base, located at coordinates 26°40’14”N, 106°37’20”E. All materials were managed according to standard agricultural practices, which included fertilization, irrigation, and weeding. To ensure seed purity, three panicles exhibiting uniformity were selected form each variety before flowering, and bagged self-pollination was facilitated using sulfuric acid paper bags. Upon harvest, grain samples were immediately frozen in liquid nitrogen to instantaneously halt all biological activities and preserve the *in vivo* state of metabolites and transcripts. The frozen samples were then stored at -80°C until further analysis.

### 2.2 Carotenoid extraction and determination

The carotenoid extraction method was carried out with reference to Ma et al. (2017). Mature sorghum seeds, preserved at -80°C, were pulverized into a fine powder using a tissue grinder (30 Hz, 1 min). Three biological replicates were analyzed for each sample, and each biological replicate was measured once. A 0.05 g portion of this powder was transferred into a centrifuge tube, to which 0.5 mL of a solvent mixture comprising n-hexane/acetone/ethanol (1:1:1, v/v/v) containing 0.01% 2, 6-ditert-butyl-4-methylphenol (BHT), was added. The mixture was vortexed at room temperature for 20 min and subsequently centrifuged at 12, 000 rpm for 5 min in a 4°C. The supernatant was carefully transferred into a new centrifuge tube. The same volume of extraction solution was added again to the original tube, and the extraction process was repeated. The supernatants from both extractions were combined. The pellet was then concentrated to dryness using a vacuum concentrator and reconstituted in 100 μL of a mixed solution of methanol/methyl tert-butyl ether (1:1, v/v). Following filtration through a 0.22 μm membrane filter, the sample was stored in a brown sample vial for UPLC-MS/MS analysis.

Carotenoids were quantified using ultra-high-performance liquid chromatography (ExionLC™ AD, https://sciex.com.cn/) coupled with tandem mass spectrometry (QTRAP^®^ 6500+, https://sciex.com.cn/) (UPLC-MS/MS), according to the methodologies reported by Krinsky et al. (2004) and Geyer et al. (2004). A YMC C30 chromatographic column was employed for liquid phase analysis, with an injection volume of 2 μL. The binary mobile phase comprised methanol/acetonitrile (1:3, v/v) (phase A) with 0.01% BHT and 0.01% formic acid, and methyl tert-butyl ether (phase B) with 0.01% BHT. The linear elution gradient was programmed as follows: 0 min, 100% A; 3 min, 100% A; 5 min, 30% A + 70% B; 9 min, 5% A + 95% B; 10 min, 100% A; 11 min, 100% A. The flow rate was maintained at 0.8 mL/min, and the column temperature was set at 28°C. Mass spectrometry conditions included an atmospheric pressure chemical ionization (APCI) source at 350°C, and a curtain gas pressure of 25 psi. All standard compounds, such as α-carotene, lycopene, γ-carotene, used for carotenoid identification and quantification were procured from Sigma-Aldrich. A metabolite database was established using these standard compounds to facilitate the qualitative analysis of the mass spectrometry detection data. Quantification Strategy:1) Full Quantification: For carotenoids with commercially available authentic standards (e.g., β-carotene, lutein, zeaxanthin), a complete method validation was conducted, including the determination of the lower limit of quantification (LLOQ) and upper limit of quantification (ULOQ). 2) Semi-Quantification: For carotenoid derivatives (e.g., acylated xanthophylls) lacking commercial standards, a semi-quantitative approach was employed. This approach utilized the calibration curve of the closest structural analogue, a well-established practice in comparative carotenoid – targeted metabolomics. Additionally, a standard curve was plotted using the multiple reaction monitoring (MRM) mode of triple quadrupole mass spectrometry to quantify the concentration of the detected metabolites.

### 2.3 RNA extraction and gene expression analysis

Mature grains samples (30 days post-anthesis) from five sorghum varieties were collected, with three biological replicates per sample. Subsequently, transcriptome libraries were constructed using the NEBNext^®^Ultra™ RNA Library Prep Kit. Following quality assessment, sequencing was conducted on the NovaSeq 6000 platform using a paired-end sequencing strategy. The sequencing data underwent quality control via Trimmomatic software (Kim et al., 2019) to eliminate adapters, and the resulting clean data were aligned to the Hongyingzi reference genome. Gene expression levels were quantitatively assessed using Cufflinks software (Trapnell et al., 2012) and expressed as FPKM (Fragments Per Kilobase of transcript per Million mapped reads). Differentially expressed genes (DEGs) involved in the grain carotenoid synthesis pathway were identified and analyzed across pairwise comparisons of sorghum grains of different colors using the DESeq2 v1.22.1 software package (Varet et al., 2016). The criteria for DEG screening included a false discovery rate (FDR) < 0.05 and an absolute log2 fold change ≥ 1. The identified DEGs were further subjected to KEGG (Kyoto Encyclopedia of Genes and Genomes) pathway enrichment analysis. To investigate transcriptional changes associated specifically with carotenoid accumulation, all varieties were compared against the material HYZ, exhibiting the lowest carotenoid content. This variety served as a biological baseline, allowing the identification of DEGs and metabolic pathways activated as carotenoid levels increased among the other varieties. This comparison strategy enhances the sensitivity of detecting carotenoid-related transcriptional regulation while reducing interference from other pigment classes.

### 2.4 Integrated metabolome and transcriptome analysis

An enrichment analysis was conducted utilizing the hypergeometric test, with pathways from the KEGG database (https://www.genome.jp/kegg) serving as the analytical units. This test was employed to identify pathways that were significantly enriched among differentially expressed metabolites and genes, in comparison to the entire genomic background (Kanehisa et al., 2008). Correlation analysis was conducted using the quantitative values of genes and metabolites across all samples. The Pearson correlation coefficient between genes and metabolites was calculated using the cor function in R. Differentially expressed genes and metabolites exhibiting a correlation coefficient greater than 0.80 and a p-value less than 0.05 were selected for further analysis.

## 3 Results

### 3.1 Carotenoid-targeted metabolomic analysis of sorghum grains

#### 3.1.1 Diversity analysis of carotenoid content in five colors grains

**Figure 1:**
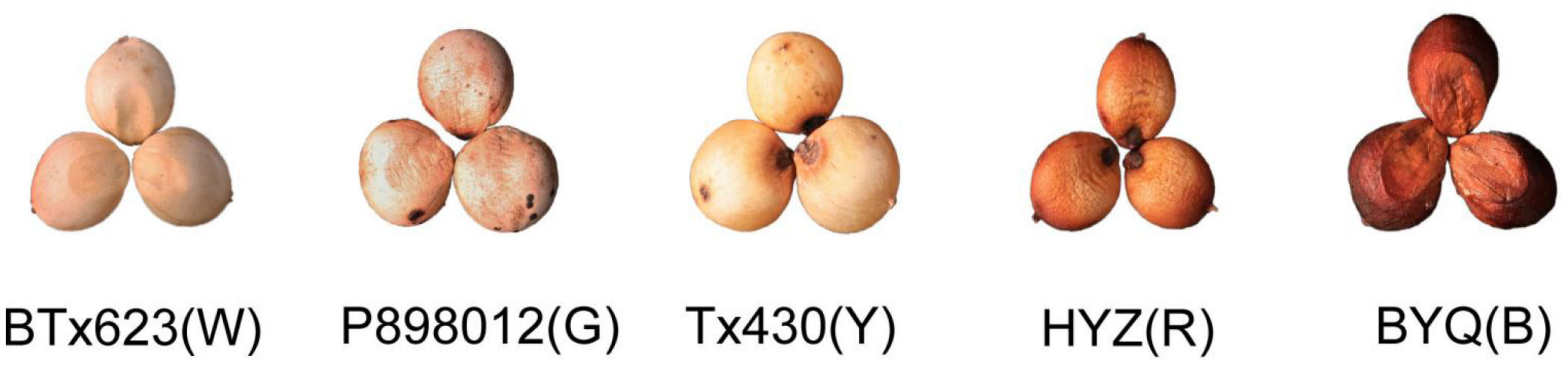
Grain phenotypes of the five sorghum grains with distinct colors. Mature grains of the five sorghum varieties(30 days post-anthesis) are displayed against a white background. From left to right: BTx623(W), the sorghum varieties BTx623 with white color, P898012(G): the sorghum varieties P898012 with gray color; Tx430(Y): the sorghum varieties Tx430 with yellow color; HYZ(R): the sorghum varieties HYZ with red color; BYQ(B): the sorghum varieties BYQ with black color.

**Figure 2:**
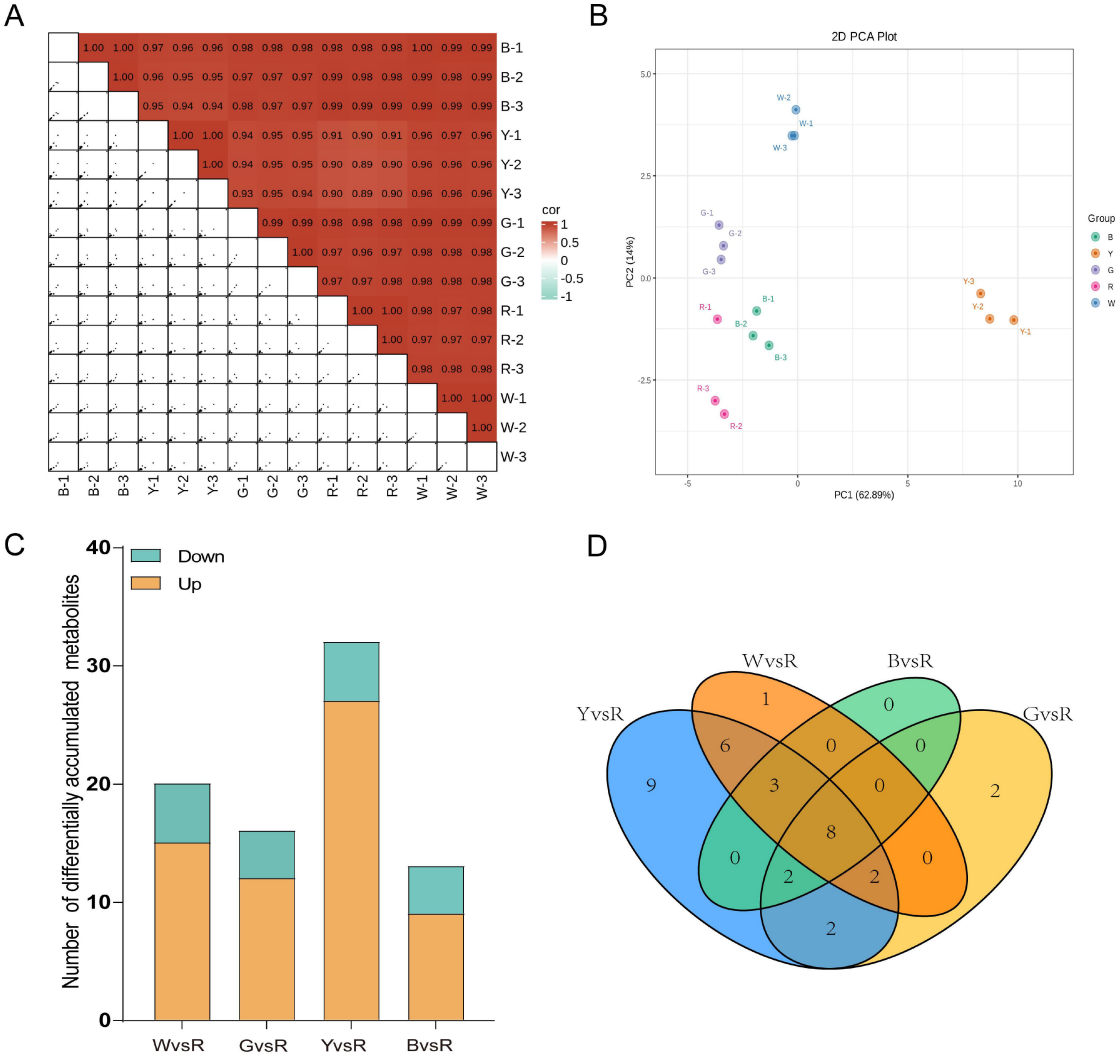
Comprehensive carotenoid-targeted metabolomics analysis of sorghum grains with five distinct colors. **(A)** Correlation coefficients between metabolites data sets from three biological duplicates and different sorghum varieties. The color scale represented correlation coefficients ranging from -1 to 1. Label sample IDs (e.g., B-1, B-2, B-3) on both axes. The diagonal line of identity showed perfect self-correlation (r = 1). High correlation coefficients within biological replicates for each variety demonstrate the high reproducibility of the metabolomic data. **(B)** Principal component analysis between metabolites data sets based on three biological duplicates and different sorghum varieties. Plot the first two principal components (PC1 and PC2) to visualize sample clustering. The dispersion of sample R1 from its biological replicates likely reflects the natural biological variability inherent to the open-pollinated landrace variety. **(C)** Bar graphs showing the upregulated and downregulated metabolites in four pairwise comparisons (W vs. R, G vs. R, Y vs. R, B vs. R), with the red variety (R) used as the common reference. **(D)** Venn diagram showing differentially metabolites (DMs) between each pairwise comparison. The distinct colored ellipses represent the unique and common DEG sets for each comparison..

In order to elucidate the carotenoid content and composition in sorghum grains of five distinct colors, we employed UPLC-MS/MS to determine these parameters (**Figure 1**, **Table 1**). The UPLC-MS/MS method was rigorously validated for each carotenoid standard. Calibration curves were established with appropriate weighting (1/x²), all showing excellent linearity (r > 0.99). The Lower Limit of Quantification (LLOQ) and Upper Limit of Quantification (ULOQ) were determined for each compound (**Supplementary Table S1**). Correlation analysis showed that the correlation coefficient between biological replicates of the same variety exceeded 0.9 (**Figure 2A**), indicating high reproducibility among the biological replicates. Furthermore, principal component analysis (PCA) identified significant variations among sorghum grain samples of different colors (**Figure 2B**).

**Table 1:** Carotenoid content in five sorghum varieties grains.

Class	Carotenoid (μg/g DW)	W	G	Y	R	B
Carotenes	β-carotene	0.2074±0.0161b	0.0901±0.0123c	0.3223±0.0031a	0.0490±0.0077d	0.0957±0.0108c
	ϵ-carotene	–	–	–	0.0032±0.0028a	–
	phytoene	–	0.2255±0.0070a	0.1152±0.0227b	–	–
xanthophylls	zeaxanthin-myristate-palmitate	–	–	0.0050±0.0006a	–	–
	zeaxanthin-laurate-myristate	–	–	0.0076±0.0028a	–	–
	zeaxanthin myristoleate	0.0121±0.0008a	–	–	–	–
	lutein myristate	0.1157±0.0084b	0.0594±0.0041c	0.2496±0.0131a	0.0388±0.0107d	0.1119±0.0038b
	lutein palmitate	0.0531±0.0100c	0.0353±0.0078d	0.1571±0.0043a	0.0547±0.0095c	0.0731±0.0086b
	lutein dimyristate	0.3767±0.0247b	0.0619±0.0049d	1.4115±0.0250a	0.1190±0.0171c	0.3930±0.0258b
	lutein oleate	0.0238±0.0048c	0.0341±0.0077bc	0.0365±0.0069bc	0.0475±0.0221ab	0.0630±0.0016a
	violaxanthin laurate	0.0264±0.0034b	0.0080±0.0011c	0.0490±0.0099a	0.0099±0.0013c	0.0211±0.0031b
	violaxanthin myristate	0.6549±0.0419a	0.5459±0.0450c	0.6170±0.0528ab	0.6162±0.0404ab	0.6538±0.0703a
	zeaxanthin	1.1580±0.0513b	0.7735±0.1151c	3.5244±0.1954a	0.3106±0.0434d	0.6668±0.0761c
	violaxanthin	0.1539±0.0095a	0.0388±0.0080c	0.0984±0.0059b	0.0424±0.0076c	0.0523±0.0098c
	neoxanthin	0.2194±0.0067a	0.0710±0.0088c	0.2152±0.0047a	0.0560±0.0091c	0.0965±0.0120b
	lutein	4.3329±0.0844b	2.3351±0.2009cd	6.7396±0.2931a	1.8929±0.2615d	2.6149±0.3253c
	β-cryptoxanthin	0.0679±0.0056b	0.0348±0.0066c	0.0793±0.0020a	0.0272±0.0041c	0.0325±0.0032c
	echinenone	0.0006±0.0001b	0.0005±0.0000b	0.0009±0.0001a	0.0006±0.0002b	0.0006±0.0001b
	lutein dilaurate	0.4013±0.0262b	0.0637±0.0125d	1.2902±0.0487a	0.1363±0.0100c	0.3709±0.0517b
	lutein dipalmitate	0.0607±0.0105c	0.0130±0.0023d	0.5266±0.0313a	0.0573±0.0156c	0.0969±0.0116b
	zeaxanthin dilaurate	–	–	0.0118±0.0025a	–	–
	violaxanthin-myristate-caprate	0.1774±0.0072b	0.0683±0.0132d	0.4001±0.0368a	0.1308±0.0259c	0.1720±0.0183bc
	antheraxanthin	0.1052±0.0053a	0.0130±0.0023b	0.1092±0.0027a	0.0112±0.0105c	0.0195±0.0043c
	5,6epoxy-luteincaprate-palmitate	0.0170±0.0148ab	0.0360±0.0032ab	0.0111±0.0098b	0.0373±0.0325ab	0.0483±0.0087a
	zeaxanthin dimyristate	0.0037±0.0003bc	–	0.0450±0.0037a	0.0049±0.0025b	0.0059±0.0017b
	lutein caprate	0.0099±0.0013b	–	0.0254±0.0045a	0.0042±0.0040c	0.0136±0.0018b
	lutein stearate	0.0110±0.0100	0.0155±0.0084	0.0201±0.0032	0.0208±0.0090	0.0155±0.0057
	violaxanthin dilaurate	0.0523±0.0071b	0.0241±0.0050c	0.1378±0.0116a	–	–
	zeaxanthin dipalmitate	–	–	0.0232±0.0030a	0.0052±0.0049bc	0.0103±0.0030b
	canthaxanthin	0.0001±0.0001ab	–	–	0.0002±0.0001a	0.0001±0.0001ab
	β-cryptoxanthin laurate	0.0100±0.0023ab	–	0.0263±0.0008a	–	0.0112±0.0008ab
	α-cryptoxanthin	–	–	0.0130±0.0012a	–	–
	capsanthin	–	–	–	0.0487±0.0010a	–
	lutein dioleate	–	–	0.0228±0.0045a	–	–
	violaxanthin-myristate-laurate	–	–	0.4152±0.0758a	–	–
	violaxanthin dimyristate	–	–	0.0402±0.0143a	–	–
	violaxanthin-myristate-palmitate	0.0272±0.0040b	0.0192±0.0016c	0.0499±0.0029a	0.0435±0.0048a	0.0464±0.0057a
	Total	8.2928±0.0449b	4.5856±0.1905d	16.8020±0.6077a	3.7987±0.3748e	5.6859±0.4621c

Data are presented as mean ± SD (n=3). For each carotenoid, different lowercase letters a, b, c, and d in a row indicated significances among means (P< 0.05); SD represents standard deviation calculated from biological replicates. For technical replicates, no repeat measurements were performed, and thus, only biological replicates (n=3) were used for statistical analysis. The quantitative data are derived from two approaches: (1) For compounds with available authentic standards (e.g., β-carotene, lutein, zeaxanthin), values represent absolute concentrations determined from their own calibration curves. (2) For carotenoid derivatives without commercial standards (e.g., esterified xanthophylls), concentrations are semi-quantitative estimates based on the calibration curve of the closest structural analogue (see **Supplementary Table S1** for the complete mapping).

A comprehensive analysis of five sorghum varieties revealed the presence of 31 carotenoids, which serve as intermediates in the carotenoid biosynthesis pathway. Of these, three were classified as carotenes, while the remaining 29 were identified as xanthophylls. Significant variations were observed in both the composition and the total carotenoid content across the different sorghum varieties (**Table 1**). Among the varieties, Y exhibited the highest number of detected carotenoid components totaling 33. This was followed by varieties W and R (HYZ), each with 26 components, and variety B with 25 components. The G variety had the fewest detected components, with only 22. Notably, 20 of these carotenoids were consistently detected across all five varieties. Seven unique carotenoids were exclusively identified in the Y variety, including zeaxanthin-myristate-palmitate, zeaxanthin-laurate-myristate, zeaxanthin dilaurate, α-cryptoxanthin, lutein dioleate, violaxanthin-myristate-laurate, and violaxanthin dimyristate. Additionally, two carotenoids, ϵ-carotene and capsanthin, were exclusively detected in the R variety, while zeaxanthin myristoleate was uniquely identified in the W variety.

Significant variations were observed in the carotenoid species content among sorghum lines of different colors (p < 0.05, **Table 1**). It is noteworthy that for certain carotenoids detected at very low abundances (e.g., echinenone), their concentrations fell below the LLOQ. While the absolute quantitative values for these compounds should be interpreted with caution, their consistent detection across biological replicates allows for reliable assessment of their relative differences among the sorghum varieties, which is the primary focus of this study. The yellow line (Y) exhibited the highest total carotenoid content, measuring 16.8020 ± 0.6077 μg/g, which was significantly higher than that of the other four lines. This was followed by the white line(W) (8.2928 ± 0.0449 μg/g), the black line(B) (5.6859 ± 0.4621 μg/g), and the gray line(G) (4.5856 ± 0.1905 μg/g). The red R line demonstrated the lowest carotenoid content, recorded at 3.7987 ± 0.3748 μg/g. Lutein was the predominant carotenoid across all lines, comprising 52.34%, 51.12%, 40.15%, 50.23%, and 46.00% of the total carotenoids in W, G, Y, R, and B, respectively. These findings showed that carotenoid content is not the primary determinant of the color in white, gray, red, and black sorghum grains, although it is notably highest in yellow grains.

#### 3.1.2 Analysis of differential carotenoid metabolites in five colors grains

Through a comprehensive carotenoid-targeted metabolomic analysis of sorghum lines exhibiting various colors, we identified metabolites that were significantly up or down-regulated were identified based on the screening criteria of variable importance in projection (VIP) ≥ 1, Fold_Change ≥ 2, or Fold_Change ≤ 0.5. Notably, no common differential carotenoid metabolites were observed across among the samples of the five distinct colors. In comparison to the R line, which exhibited the lowest carotenoid content, 20 differential metabolites were identified between W and R, with 15 up-regulated and 5 down-regulated, primarily enriched in 5 KEGG pathways. Between G and R, 16 differential metabolites were identified, with 12 up-regulated and 4 down-regulated, enriched in 4 KEGG pathways. The Y and R lines exhibited the highest number of differential metabolites, totaling 32, with 27 up-regulated and 5 down-regulated, mainly enriched in 5 KEGG pathways. Conversely, the number of differential metabolites between B and R was the lowest, with 13, including 9 up-regulated and 4 down-regulated, enriched in 4 KEGG pathways (**Figure 2C**). Across these comparisons, 8 common differential metabolites were identified (**Figure 2D**).

### 3.2 Transcriptomic analysis of five colors grains

#### 3.2.1 Overview of transcriptomic profiling and differential expression analysis

**Figure 3:**
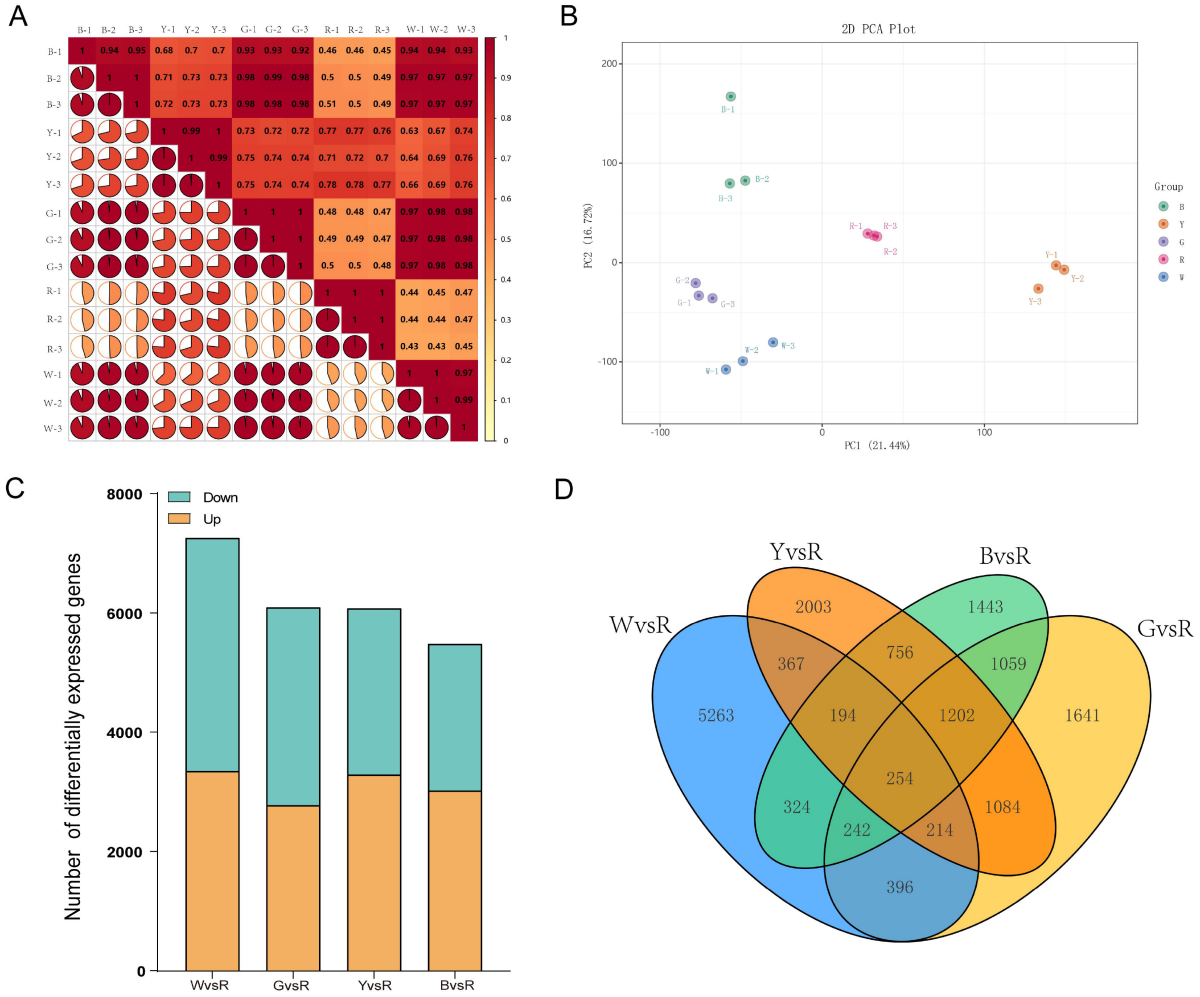
Gene expression profiling of five samples. **(A)** Correlation coefficients between gene expression data sets from three biological duplicates and different sorghum varieties. The heatmap displays Pearson correlation coefficients (color scale: -1 to 1), with darker red indicating higher reproducibility. Label sample IDs (e.g., B-1, B-2, B-3) on both axes. High correlation coefficients within each variety’s replicates confirm the reliability of the transcriptomic data. **(B)** Principal component analysis between gene expression data sets based on three biological duplicates and different sorghum varieties. Plot the first two principal components (PC1 and PC2) to visualize sample clustering. **(C)** Bar graphs showing the upregulated and downregulated metabolites in four pairwise comparisons (W vs. R, G vs. R, Y vs. R, B vs. R), with the red variety (R) used as the common reference. **(D)** Venn diagram showing differentially expressed genes between each pairwise comparison. The distinct colored ellipses represent the unique and common DEG sets for each comparison.

To explore the transcriptional profiles of sorghum grains, cDNA libraries were constructed from three biological replicates of each of the five sorghum grain color variants, 30 days post-anthesis, to facilitate transcriptome sequencing. Correlation analysis revealed that the correlation coefficient among biological replicates of the same v variety exceeded 0.9 (**Figure 3A**), indicating high reproducibility. Additionally, Principal Component Analysis (PCA) showed significant differentiation among the sample groups (**Figure 3B**), indicating significant transcriptional differences between sorghum grains of varying colors. The transcriptome sequencing yielded over 4.28 Gb of clean reads per sample, culminating in a total of 786, 212, 722 clean reads and 117.92 Gb of total base pairs. The GC content of sorghum grains ranged from 56.34% to 59.15%, and the Q30 base percentage surpassed 91.25% (**Supplementary Table S2**). Collectively, these findings affirm that the sequencing data’s quantity and quality are of sufficient quality for subsequent analyses. After removing adapter sequences and low-quality reads, the clean reads from each library were mapped to the reference genome (HYZ genome), which is publicly available at: https://db.cngb.org/data_resources/assembly/CNA0105939. This process identified a total of 44, 897 genes, along with 12, 229 novel transcripts that were not annotated in the reference gene set.

Based on the criteria of |log2fold change| ≥ 1 and FDR < 0.05, pairwise comparisons among the five varieties identified a total of 14, 180 differentially expressed genes (DEGs) (**Supplementary Table S3**). Among these comparisons, WvsR group exhibited the largest number of DEGs (7, 254), including 3, 343 up-regulated and 3, 911 down-regulated DEGs. There were 6, 092 DEGs between G and R, with 2, 771 up-regulated and 3, 321 down-regulated. While Y vs R comparison yielded 6, 074 DEGs, including 3, 286 up-regulated and 2, 788 down-regulated. The smallest number of DEGs was observed in B vs R, with 5, 474, with 3, 019 up-regulated and 2, 455 down-regulated (**Figure 3C**). These results indicated that the number of DEGs between white grains and red grains was the most pronounced. Venn diagram analysis identified 254 common genes across the DEGs of all four combination groups (**Figure 3D**). KEGG pathway enrichment analysis showed that the DEGs from W vs R, G vs R, Y vs R, and B vs R comparisons were associated with 134, 139, 138, and 137 KEGG pathways, respectively (**Supplementary Table S3**).

**Figure 4:**
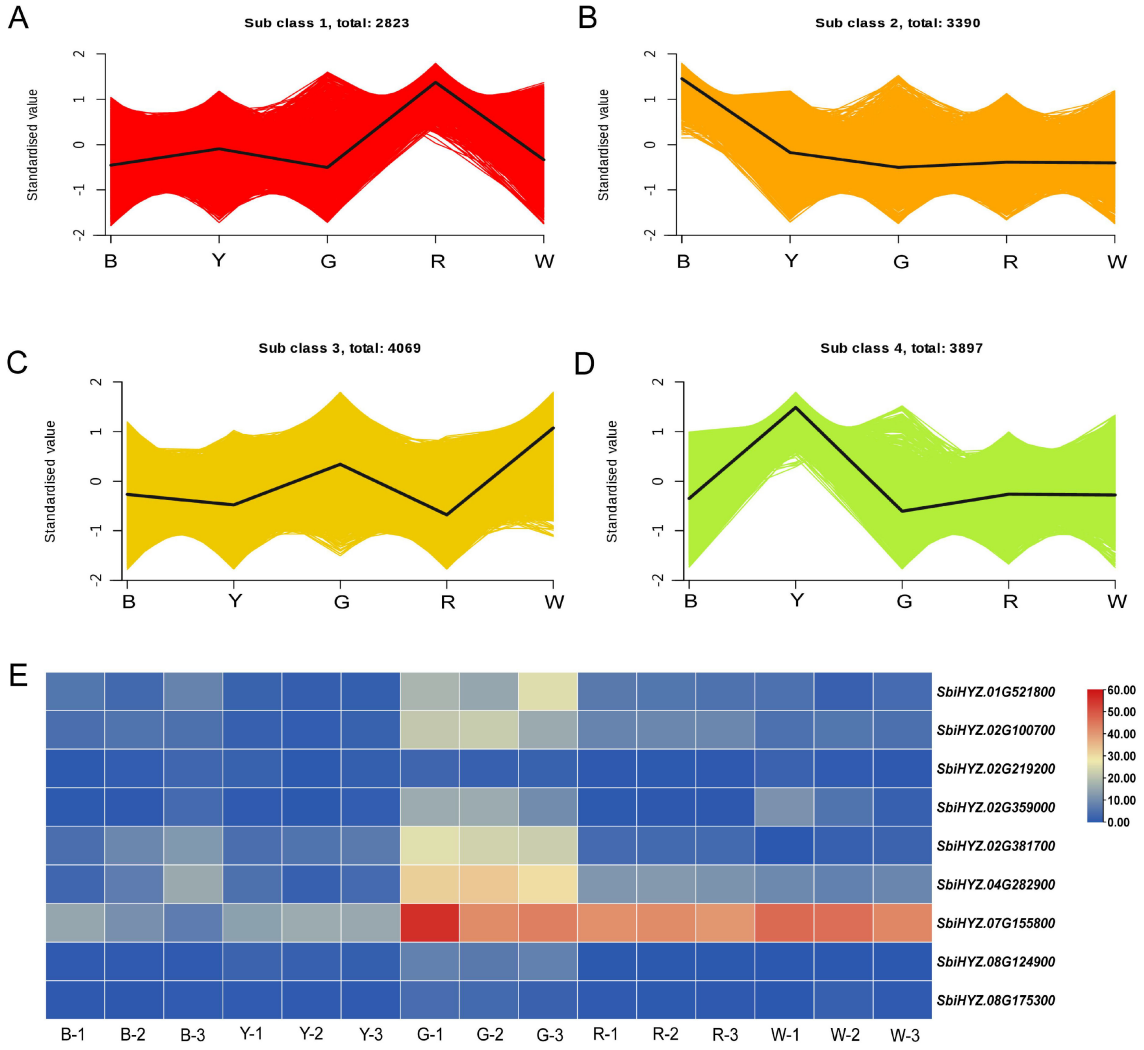
Expression trends and patterns of differentially expressed genes (DEGs) across five sorghum varieties. **(A–D)** Line graphs display the standardized expression trends of DEGs across the five sorghum varieties with four distinct subclasses (Sub class1-4). The solid line represents the average standardized expression value for all genes within the subclass, while the shaded area depicts the confidence interval or expression variability. **(E)** Heatmap illustrating the expression levels of nine key carotenoid metabolism-related genes in Sub class 4 across three biological replicates of each sorghum variety. Each row represents a gene, and each column represents a sample. The color scale from blue to red indicates normalized expression values. SbiHYZ.01G521800(3-monooxygenase), SbiHYZ.02G100700(Xanthine dehydrogenase), SbiHYZ.02G219200(Abscisic acid 8'-hydroxylase 3), SbiHYZ.02G359000(Momilactone A synthase), SbiHYZ.02G381700(15-cis-phytoene desaturase), SbiHYZ.04G282900(Abscisic acid 8'-hydroxylase 1), SbiHYZ.07G155800(Abscisic acid 8'-hydroxylase 3), SbiHYZ.08G124900(Cytochrome P450 716A1), and SbiHYZ.08G175300(9cis-epoxycarotenoid dioxygenase NCED5).

A K-means clustering analysis was conducted on the identified DEGs to categorize them based on similar variation trends. Ultimately, 14, 180 DEGs were classified into four distinct subclasses, demonstrating differential expression across various samples (**Figures 4A–D**; **Supplementary Table S4**). Through correlation analysis between gene expression patterns within these subclasses and changes in carotenoid content, genes in Subclass 4 were selected for further investigation to identify those involved in the regulation of carotenoid synthesis. The results showed that nine genes within Subclass 4 were enriched in the carotenoid biosynthesis pathway, These include: *SbiHYZ.01G521800(3-monooxygenase)*, *SbiHYZ.02G100700(Xanthine dehydrogenase)*, *SbiHYZ.02G219200(Abscisic acid 8’-hydroxylase 3)*, *SbiHYZ.02G359000(Momilactone A synthase)*, *SbiHYZ.02G381700(15-cis-phytoene desaturase)*, *SbiHYZ.04G282900(Abscisic acid 8’-hydroxylase 1)*, *SbiHYZ.07G155800(Abscisic acid 8’-hydroxylase 3)*, *SbiHYZ.08G124900(Cytochrome P450 716A1)*, and *SbiHYZ.08G175300(9-cis-epoxycarotenoid dioxygenase NCED5)*. Among these, 15-cis-phytoene desaturase catalyzes a key desaturation step converting phytoene to ζ-carotene, which is essential for the formation of downstream carotenoids. Cytochrome P450 716A1 and 3-monooxygenase are involved in oxidative modification steps, contributing to the structural diversification of carotenoid derivatives. Xanthine dehydrogenase may participate in oxidative stress responses, indirectly influencing carotenoid stability. Notably, three abscisic acid 8′-hydroxylases (encoded by *SbiHYZ.02G219200*, *SbiHYZ.04G282900*, and *SbiHYZ.07G155800*) and 9-cis-epoxycarotenoid dioxygenase NCED5 (*SbiHYZ.08G175300*) play critical roles in the degradation of carotenoids to abscisic acid (ABA), linking carotenoid metabolism to hormone biosynthesis. Momilactone A synthase may be involved in the branching of diterpenoid and carotenoid pathways. Together, these genes indicate coordinated regulation between carotenoid biosynthesis and catabolism, potentially contributing to the carotenoid-induced color differences in sorghum grains. The expression levels of eight genes were analyzed in sorghum grains of five different colors. Notably, the expression level of *SbiHYZ.07G155800* was consistently high across all samples, and all eight genes exhibited elevated expression in the gray sorghum grain G (**Figure 4E**).

#### 3.2.2 Expression profiles of carotenoid metabolism-related genes among sorghum grains of different colors

**Figure 5:**
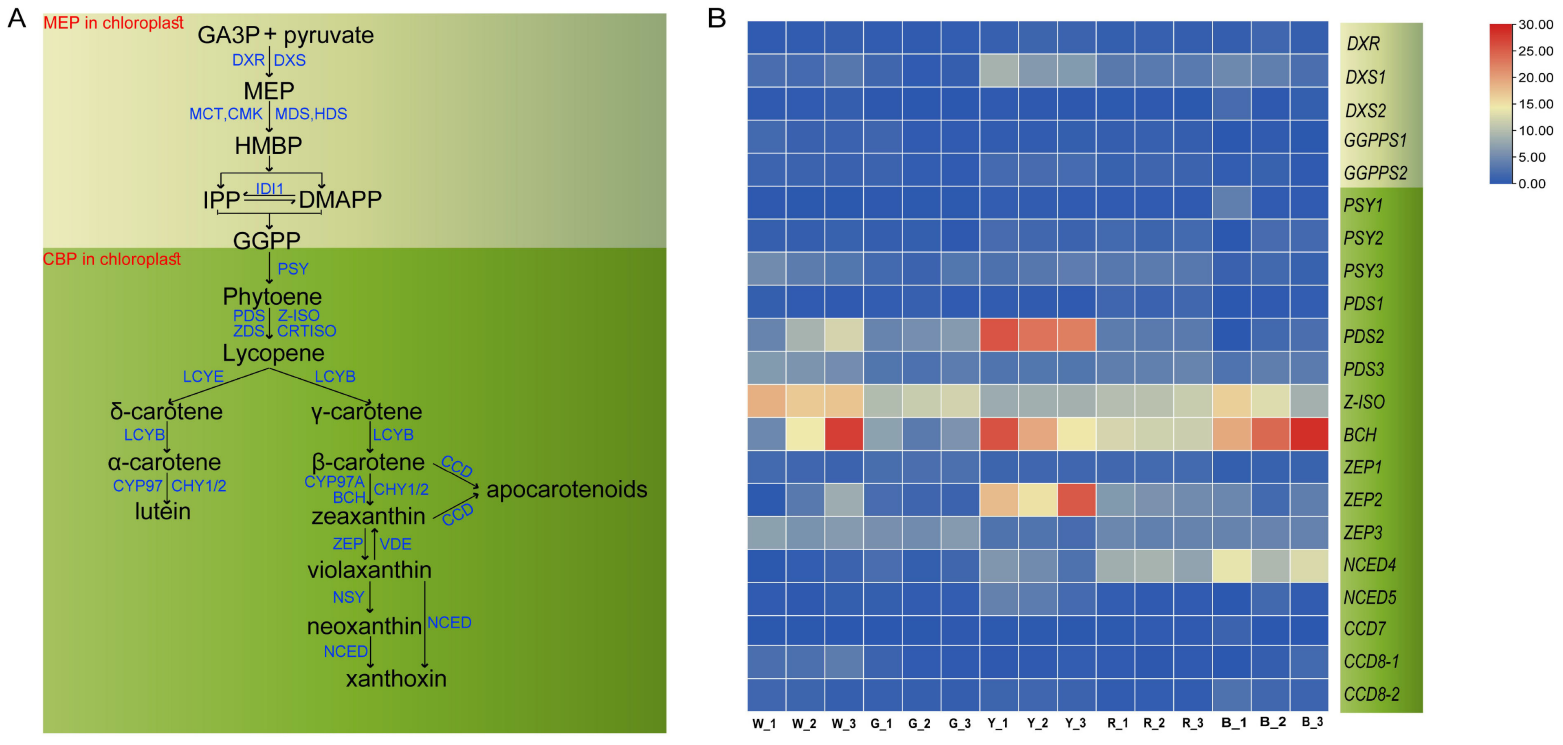
Three successive biosynthesis carotenoid metabolic pathways [MEP (2-C-methyl-D-erythritol-4-phosphate), CBP(carotenoid biosynthesis pathway)] and expression profiles of related genes in sorghum grains. **(A)** Schematic representation of the carotenoid metabolic pathway. Major intermediates and corresponding enzymes are shown, with enzyme names indicated in blue italics. **(B)** Heatmap of the expression profiles of carotenoid-related genes in sorghum grains with different pericarp colors. The color scale indicates normalized expression levels from low (blue) to high (red). W, G, Y, R, and B represent white, gray, yellow, red, and black grains, respectively, each with three biological replicates. MEP pathway. Components: GA3P, D-Glyceraldehyde 3-phosphate; Pyruvate,1-Deoxy-D-xylulose 5-phosphate; HMBP, 4-Hydroxy-3-methylbut-2-enyl-diphosphate; IPP, Isopentenyl diphosphate, C5; DMAPP, dimethylallyl diphosphate, C5; GGPP, Geranylgeranyl diphosphate, C20. Enzymes/genes:DXS, 1-Deoxy-D-xylulose 5-phosphate synthase; DXR, 1-Deoxy-D-xylulose 5-phosphate reductoisomerase; MCT, 2-C-methyl-D-erythritol 4-phosphate cytidylyltransferase; CMK, 4-(cytidine 5-diphospho)2-C-methyl-D-erythritol kinase; MDS, 2-C-methyl-D-erythritol 2,4-cyclodiphosphate synthase; HDS, 4-Hydroxy-3-methylbut-2enyl-diphosphate synthase; HDR, 4-Hydroxy-3-methylbut-2-enyl-diphosphate reductase; IDI, Isopentenyl diphosphate Δ isomerase; GGPPS, Geranylgeranyl diphosphate synthase. CBP (carotenoid biosynthesis pathway). Enzymes/genes: CCDs, carotenoid cleavage dioxygenases; CHY1, 1-ring carotene hydroxylase; CHY2, 2-ring carotene hydroxylase; CRTISO, carotene isomerase; CYP97A, β-ring hydroxylase; LCYB, lycopene β-cyclas; LCYE, lycopene ϵ-cyclase; NCEDs, 9-cis-epoxycarotenoid dioxygenases; NSY, neoxanthin synthase; PDS, phytoene desaturase; PSY, phytoene synthase; ZDS, ζ-carotene desaturase; ZEP, zeaxanthin epoxidase; ZISO, ζ-carotene isomerase.

Differences in carotenoid content among sorghum grains are regulated by the expression of key genes involved in carotenoid biosynthesis and degradation (**Figure 5A**). To further elucidate the molecular mechanisms underlying carotenoid variation, we analyzed the expression profiles of genes associated with carotenoid metabolism, including the upstream 2-C-methyl-D-erythritol 4-phosphate (MEP) pathway and downstream biosynthetic and degradation pathways (**Figure 5B**). The results revealed that multiple key genes exhibited distinct expression patterns among white (W), gray (G), yellow (Y), red (R), and black (B) sorghum varieties (**Figure 5B**), indicating that differential transcriptional regulation of carotenoid metabolism may contribute to grain color diversity.

In the upstream MEP pathway, genes involved in precursor synthesis, such as *DXS*(1-deoxy-D-xylulose-5-phosphate synthase) and *GGPPS*(geranylgeranyl diphosphate synthase), showed higher expression levels in yellow grains than in light-colored grains (W and G). The elevated expression of these genes likely enhances the production of carotenoid precursors, providing ample substrate for downstream pigment biosynthesis. Within the core carotenoid biosynthesis pathway, members of the *PSY*(phytoene synthase) gene family, particularly *PSY1*and *PSY2*, were markedly upregulated in red and black grains. Since PSY is a rate-limiting enzyme in carotenoid biosynthesis, its increased expression is generally correlated with enhanced pigment accumulation. In addition, *PDS*(phytoene desaturase), which catalyzes the conversion of phytoene to lycopene, exhibited higher expression in yellow grains than in light-colored varieties, indicating a more efficient synthesis of colored carotenoid intermediates. Among three homologs-*PDS1*, *PDS2*, and *PDS3*-*PDS2(SbiHYZ.02G381700)* exhibited the most significant differential expression, as indicated by the expression patterns in **Figure 4E** and **Figure 5B**. These findings suggest that *PDS2* primarily contributes to differential carotenoid accumulation in sorghum grains. Regarding carotenoid modification and degradation, *BCH*(β-carotene hydroxylase) was more highly expressed in yellow and red grains, suggesting a more active conversion of β-carotene to zeaxanthin. Similarly, degradation-related genes such as *ZEP*(zeaxanthin epoxidase) and *NCED*(9-cis-epoxycarotenoid dioxygenase) showed elevated transcript levels in yellow and red grains, implying enhanced metabolic flux toward xanthophyll derivatives such as violaxanthin and apocarotenoids. These results indicate that, in addition to variations in biosynthetic capacity, the branching and turnover of carotenoid pathways also differ among sorghum varieties. It should be noted that sorghum grain color is not determined solely by carotenoids but is also influenced by other pigments such as anthocyanins and phenolic compounds. Therefore, the differential expression of carotenoid-related genes identified in this study represents an important, though not exclusive, regulatory layer contributing to grain color diversity in sorghum.

### 3.3 Integrated transcriptome and carotenoid-targeted metabolome analysis

The integrated transcriptomic and carotenoid-targeted metabolomic analysis showed that the identified DEGs and differential metabolites were predominantly enriched in five KEGG pathways: Carotenoid biosynthesis (ko00906), Metabolic pathways (ko01100), Biosynthesis of secondary metabolites (ko01110), Biosynthesis of various plant secondary metabolites (ko00999), and Biosynthesis of cofactors (ko01240). Specifically, the Carotenoid biosynthesis pathway (ko00906) was enriched with three differential metabolites (β-carotene, zeaxanthin, lutein) and two DEGs [*SbiHYZ.02G381700* (*PDS*), *SbiHYZ.08G124900* (*CYP716A1*)] (**Table 2**). Correlation analysis between the differential metabolites and DEGs indicated that *PDS* and *CYP716A1* may positively regulate the differential metabolites (β-carotene, zeaxanthin, lutein) within the carotenoid metabolic pathway (P<0.05). Notably, no differential carotenoids or genes were enriched in B vs R comparison, potentially due to both varieties exhibiting red coloration, where anthocyanins in sorghum grains predominantly influence color formation.

**Table 2:** Statistics of differential metabolites and differentially expressed genes related to the carotenoid synthesis pathway in sorghum grains of different colors.

Group	Differential metabolites	Differential genes	Description	Pathways	p-value	Correlation coefficient
W vs R	β-carotene	*SbiHYZ.02G381700*	*15-cis-phytoene desaturase*	ko00906; ko01100; ko01110	0.1571	0.81
	zeaxanthin	*SbiHYZ.02G381700*	15-cis-phytoene desaturase	ko00906; ko01100; ko01110	0.1571	0.88
	lutein	*SbiHYZ.02G381700*	15-cis-phytoene desaturase	ko00906; ko01100; ko01110	0.1571	0.86
G vs R	Zeaxanthin	*SbiHYZ.08G124900*	Cytochrome P450 716A1	ko00906; ko01110	0.03329	0.85
	β-carotene	*SbiHYZ.02G381700*	15-cis-phytoene desaturase	ko00906; ko01100; ko01110	0.0876	0.81
	zeaxanthin	*SbiHYZ.02G381700*	15-cis-phytoene desaturase	ko00906; ko01100; ko01110	0.0876	0.88
Y vs R	zeaxanthin	*SbiHYZ.08G124900*	Cytochrome P450 716A1	ko00906; ko01110	0.00442	0.85
	lutein	*SbiHYZ.02G381700*	15-cis-phytoene desaturase	ko00906; ko01100; ko01110	0.00215	0.86
B vs R	*\*	*\*				

Pathway annotations: ko00906: Carotenoid biosynthesis; ko01100: Metabolic pathways; ko01110: Biosynthesis of secondary metabolites. Correlation coefficient (Pearson r) representing the association between gene expression and metabolite abundance. Positive value indicates a positive correlation.

**Table 2** Statistics of Differential Metabolites and Differentially Expressed Genes Related to the Carotenoid Synthesis Pathway in Sorghum Grains of Different Colors.

## 4 Discussion

### 4.1 Carotenoid diversity across sorghum grains of different colors, with emphasis on yellow varieties

As the fifth most important cereal crop worldwide, sorghum grains are abundant in various bioactive compounds, which demonstrate substantial biological activities, including antioxidant, anti-cancer, anti-diabetic, anti-inflammatory, and anti-obesity effects (Li et al., 2021). However, its carotenoid content is generally low compared to other cereals like maize, which limits their nutritional value in combating VAD (Cruet-Burgos et al., 2023). Carotenoids, as crucial natural pigments and antioxidants in plants, play a pivotal role in determining the coloration of cereal grains. The distribution and concentration of carotenoids in these grains directly influence their color characteristics, as well as anthocyanins, tannins, and other flavonoids (Ndolo and Beta, 2013; Tang et al., 2022). A consistent observation in sorghum is that yellow-grained varieties possess a significantly higher carotenoid content than other color varieties (McDowell et al., 2024). This study confirms that pattern, with the yellow variety (Y) exhibiting the highest carotenoid concentration. In the present study, a targeted carotenoid profiling analysis of sorghum grains of different colors revealed that the total carotenoid content in the yellow sorghum variety Y was16.8020 ± 0.6077 μg/g DW, markedly surpassing that of the white, gray, red, and black varieties, with lutein comprising 40.15% of the total carotenoids. These findings are consistent with previous research on transgenic maize, which identified the high accumulation of β-carotene and lutein are the primary contributors to the yellow grain phenotype. Furthermore, enhancing the flux toward zeaxanthin, a more deeply colored xanthophyll from the β-branch, has also been shown to effectively intensify the yellow phenotype (Aluru et al., 2008).

The contribution of carotenoid diversity to color variation is also evident in other varieties, though through different mechanisms. The red sorghum variety HYZ (R) was detected to contain unique components, ϵ-carotene and capsanthin. Despite its total carotenoid content being only 3.7987 ± 0.3748 μg/g DW, in conjunction with Ndolo and Beta (2013) study on carotenoids distribution in cereals, it can be inferred that the presence of these specific components may contribute to the red-biased coloration of the grains. It is hypothesized that these distinctive carotenoid derivatives play a unique role in the synergistic effect of pigments. It is noteworthy that no differential metabolites or genes were enriched in the carotenoid synthesis pathway when comparing the black sorghum (B) and the red sorghum (R), both of which exhibit a red coloration. This observation aligns with the findings of Ampomah-Dwamena et al. (2009) in kiwifruit, which suggested that when carotenoid levels are low, other pigments such as anthocyanins may play a more significant role in determining color.

Further analysis revealed that lutein constituted 40% to 53% of the total carotenoids across all varieties, confirming that lutein is the main form of carotenoids in crop grains. However, in non-yellow grains, there was no significant correlation between lutein content and color, suggesting that the emergence of non-yellow phenotypes may rely on the balance between carotenoids and other pigments rather than the absolute concentration of any single pigment. Additionally, although the white variety W was found to contain a unique compound, zeaxanthin myristoleate, its total carotenoid content was only 8.28 ± 0.04 μg/g DW, and no high-abundance-colored carotenoids were detected. This finding aligns with the conclusions drawn by Colasuonno et al. (2017) their study on white wheat, which posits that the development of white grains may result from the interruption of critical steps in the carotenoid synthesis pathway. This interruption leads to the accumulation of colorless precursor substances rather than indicating a total absence of carotenoid synthesis capability (Colasuonno et al., 2017).

### 4.2 Transcriptional regulatory mechanism of genes related to carotenoid synthesis

Genome-wide association studies (GWAS) have elucidated the genetic basis of carotenoid variation in sorghum, identifying multiple gene markers associated with carotenoids such as *ZEP*, *PDS*, and *CYP97A* (Cruet-Burgos et al., 2020). It is hypothesized that variations within these genes significantly contribute to the observed differences in carotenoid content among sorghum grains of different colors. Concurrently, research has demonstrated that transcriptional regulation of carotenoid biosynthesis and degradation pathways varies across different developmental stages of sorghum, thereby presenting novel molecular breeding targets for the biofortification of sorghum with carotenoids (Cruet-Burgos and Rhodes, 2023).

In the present study, transcriptomic analysis conducted in this study revealed the differential expression patterns of key genes involved in the carotenoid biosynthesis pathway, thereby providing a molecular framework for understanding the regulatory mechanism underlying sorghum grain coloration. Among the 14, 180 DEGs identified in sorghum with five colors, nine genes associated with carotenoid synthesis, including *PDS* and *CYP716A1*, were enriched in Subclass 4 and play a core role. These genes can be broadly categorized into three functional groups. First, genes like *15-cis-phytoene desaturase (PDS, SbiHYZ.02G381700)* catalyze the crucial initial desaturation of phytoene, a rate-limiting step governing carbon flux into the pathway. Second, enzymes such as *CYP716A1 (SbiHYZ.08G124900)* and *3-monooxygenase (SbiHYZ.01G521800)* are involved in oxidative modifications that generate diverse xanthophylls. Third, a significant catabolic module was identified, including *abscisic acid 8’-hydroxylases (SbiHYZ.02G219200, SbiHYZ.04G282900, SbiHYZ.07G155800)* and *9-cis-epoxycarotenoid dioxygenase NCED5 (SbiHYZ.08G175300)*, which are key enzymes in the degradation of carotenoids to abscisic acid (ABA), directly linking carotenoid turnover to hormone signaling. The expression analysis provided critical insight into the integrated regulatory impact of these genes. The concerted upregulation of all eight analyzed genes in the gray sorghum grain (G), points to a metabolic equilibrium shifted towards high turnover. This pattern suggests that the gray grain phenotype may be characterized not by a simple deficiency in biosynthesis, but by a network-based regulation where the balance between synthesis and degradation ultimately determines the final carotenoid profile.

Notably, PDS, as a rate-limiting enzyme in carotenoid synthesis, significantly enhances the accumulation of β-carotene and zeaxanthin when overexpressed (P < 0.05). In wheat, integrative analysis of transcriptomic and phenotypic data revealed that the expression level of the *PDS* gene directly influences the synthesis efficiency of downstream carotenoids (Colasuonno et al., 2017). Furthermore, the *PDS* gene mutant has impaired enzyme activity, which hinders the accumulation of carotenoid precursor substances in grains, and ultimately results in a significant lightening of grain color (Sobrino-Mengual et al., 2024). These studies substantiate the conserved and fundamental role of *PDS* in the carotenoid-mediated pigment formation process in cereals. In addition, the *CYP716A1* gene identified in this investigation exhibits a specific regulatory role in zeaxanthin synthesis, and its function is similar to that of the β-carotene hydroxylase gene as reported by Yamaguchi (2012). β-carotene hydroxylase is a pivotal enzyme responsible for hydroxylation modification within the carotenoid biosynthetic pathway, thereby reinforcing the conservation of this gene in carotenoid hydroxylation modification (Yamaguchi, 2012; Virk et al., 2021).

The integrated carotenoid-targeted metabolomic and transcriptomic analyses demonstrate that the regulatory functions of *PDS* and *CYP716A1* are specific to the variety: in the comparison between white-grained W and red-grained R, PDS exclusively regulates β-carotene synthesis; whereas in the comparison between yellow vs. red (Y vs. R), both genes synergistically regulate zeaxanthin accumulation. This genotype-dependent regulatory pattern expands the understanding that the regulatory pattern of carotenoid synthesis genes in maize is not fixed-instead, they are shaped by the genetic background of varieties, leading to the formation of divergent regulatory networks (Aluru et al., 2008). In addition, in the gray variety G, despite the high expression of eight carotenoid biosynthesis-related genes, the carotenoid content remained at a moderate level. The apparent inconsistency between high gene expression and low carotenoid content implies that additional post-transcriptional regulatory mechanisms are at play. These may involve the action of carotenoid-cleavage dioxygenases (CCDs)—which, as demonstrated by Ndolo and Beta (2013), can degrade carotenoids and reduce pigment accumulation—or potential antagonistic interactions with competing pathways such as anthocyanin biosynthesis. At the same time, the identification of 254 common differential genes indicates the presence of a universal pigment biosynthesis pathway in sorghum grains. Meanwhile, the discovery of novel transcripts provides clues for the unknown regulatory pathways of carotenoid synthesis. This observation echoes the conclusion from wheat research, where genes associated with carotenoid transport were shown to influence the distribution of grain pigments. Collectively, these results highlight the need for further exploration of uncharacterized regulatory elements to fully decipher the carotenoid metabolic network in sorghum grains.

This study employed a combination of transcriptomic and carotenoid-targeted metabolomic analyses to systematically compare the carotenoids accumulation and gene expression across five distinct sorghum varieties, advancing our understanding of the regulatory network underlying grain coloration. Nonetheless, this study still has limitations. Primarily, it focused exclusively on samples at the mature stage (30 days post-anthesis), which precludes the observation of dynamic changes in carotenoid synthesis over time. Carotenoid synthesis is a complex biological process affected by a variety of environmental factors (López–Ráez et al., 2008; Lado et al., 2019). In addition, carotenoid synthesis may also be regulated by specific developmental stages and gene expression patterns, leading to different metabolic characteristics in sorghum across different growth stages. To enhance the understanding of regulatory networks, a multi-stage analytical approach is necessary. Therefore, a primary objective for our future research will be to implement this multi-stage approach, conducting a high-resolution time-course analysis to delineate the temporal dynamics of carotenoid metabolism throughout sorghum grain development. For instance, research on wheat grain development has demonstrated that the expression levels of carotenoid synthesis genes peak during the filling stage (Colasuonno et al., 2017). Similarly, transcriptomic analysis in avocados have identified substantial variations in the expression of carotenoid synthesis-related genes across different developmental stages (Félix et al., 2018). Furthermore, the integration of omics data related to other pigments, such as anthocyanins, remains incomplete, which impedes a comprehensive understanding of the synergistic mechanisms underlying color formation. At the application level, the key genes identified in this study, including *PDS* and *CYP716A1*, hold potential as molecular markers for breeding sorghum varieties with enhanced carotenoid content. For example, the incorporation of a highly expressed *PDS* allele from variety Y into white, tannin-free sorghum varieties with desirable taste profiles is anticipated to increase carotenoid content and altering grain color. This is consistent with the strategy of improving wheat carotenoid content through genetic engineering methods (Rodríguez-Suárez et al., 2023; Palombieri et al., 2025). Furthermore, investigating the synergistic regulatory mechanisms between carotenoids and anthocyanins can provide theoretical support for developing sorghum of multi-colored grain varieties, thereby contributing to the simultaneous improvement of both the nutritional and aesthetic qualities of crops.

## 5 Conclusions

This study employed integrated carotenoid-targeted metabolomic and transcriptomic analyses to uncover the multi-omics factors behind carotenoid accumulation in sorghum grains of different colors. We achieved precise quantification of carotenoid components across five sorghum grains color variants. Among these, the yellow-grained variety (TX430) exhibited the highest carotenoid content, while the red-grained variety (HYZ) accumulated unique metabolites like ϵ-carotene. Crucially, our findings identified that the coordinated expression of the *PDS* and *CYP716A1* genes is crucial for regulating core carotenoid content, explaining transcriptional mechanisms behind color differences. By establishing a direct connection between the carotenoid metabolic profile and the dynamic transcriptional network of key biosynthesis genes, this study provides a comprehensive explanatory framework for the mechanism underlying pigment formation in sorghum grains. Furthermore, this research holds substantial value for breeding applications, as the identified key genes (e.g., *PDS*, *CYP716A1*) serve as direct targets for marker-assisted selection. This enables the rapid development of new sorghum varieties with enhanced carotenoid biofortification. For example, the introgression of elite high-expression alleles from yellow-grained sorghum into white- or red-grained varieties with superior agronomic traits is anticipated to synergistically enhance grain nutritional quality through increased carotenoid content and improve visual appearance, without the need for introducing exogenous genes. This strategy leverages the crop’s intrinsic genetic potential to offer a sustainable solution for fundamentally alleviating vitamin A deficiency.

## Data Availability

The raw RNA-seq data generated in this study have been deposited in the China National GeneBank DataBase (CNGBdb) under accession number CNP0002968. The data can be accessed at: https://db.cngb.org/data_resources/?query=CNP0002968.
